# Selective Attention Increases Both Gain and Feature Selectivity of the Human Auditory Cortex

**DOI:** 10.1371/journal.pone.0000909

**Published:** 2007-09-19

**Authors:** Jaakko Kauramäki, Iiro P. Jääskeläinen, Mikko Sams

**Affiliations:** Laboratory of Computational Engineering, Helsinki University of Technology, Espoo, Finland; University of Sydney, Australia

## Abstract

**Background:**

An experienced car mechanic can often deduce what's wrong with a car by carefully listening to the sound of the ailing engine, despite the presence of multiple sources of noise. Indeed, the ability to select task-relevant sounds for awareness, whilst ignoring irrelevant ones, constitutes one of the most fundamental of human faculties, but the underlying neural mechanisms have remained elusive. While most of the literature explains the neural basis of selective attention by means of an increase in neural gain, a number of papers propose enhancement in neural selectivity as an alternative or a complementary mechanism.

**Methodology/Principal Findings:**

Here, to address the question whether pure gain increase alone can explain auditory selective attention in humans, we quantified the auditory cortex frequency selectivity in 20 healthy subjects by masking 1000-Hz tones by continuous noise masker with parametrically varying frequency notches around the tone frequency (*i.e.*, a notched-noise masker). The task of the subjects was, in different conditions, to selectively attend to either occasionally occurring slight increments in tone frequency (1020 Hz), tones of slightly longer duration, or ignore the sounds. In line with previous studies, in the ignore condition, the global field power (GFP) of event-related brain responses at 100 ms from the stimulus onset to the 1000-Hz tones was suppressed as a function of the narrowing of the notch width. During the selective attention conditions, the suppressant effect of the noise notch width on GFP was decreased, but as a function significantly different from a multiplicative one expected on the basis of simple gain model of selective attention.

**Conclusions/Significance:**

Our results suggest that auditory selective attention in humans cannot be explained by a gain model, where only the neural activity level is increased, but rather that selective attention additionally enhances auditory cortex frequency selectivity.

## Introduction

The neural basis of selective attention constitutes one of the most fundamental questions in cognitive neuroscience and psychology. Based on previous studies in sensory systems, two alternative neural mechanisms, multiplicative increase in neural gain [1]–[16] *vs.* enhanced feature selectivity of the neurons in sensory cortices [17]–[21], have been suggested to underlie selective attention. Additionally, there are theories according to which selective attention activates neural populations separate from those processing the stimuli [15], [22].

When referring to increase in response gain in the literature, terms like ‘higher’ or ‘increased’ activity are typically used to describe the observed attentional enhancement in the modality-specific sensory cortices in human neuroimaging studies. Few studies so far have addressed the issue of whether this is simple gain change (amplification) in respective sensory areas [23] or whether there are some selective changes as well. For example, increase in activity level has been reported in functional Magnetic Resonance Imaging (fMRI) [7], [11], [15], [16], positron emission tomography (PET) [9], [10], [12], [13], electroencephalography (EEG) [1], [2], [4], [6], and magnetoencephalography (MEG) [3], [5], [8], [14] studies in humans, but the issue of whether there is a ‘bias’ mechanism in addition to the attentional gain control has been only recently raised [20].

Typically auditory selective attention studies have employed a dichotic listening task, instructing subjects to listen to one ear and ignore the sounds coming from the other (‘attend towards’ *vs.* ‘attend away’). This way, selective attention has been found to modulate neurophysiological responses to tones 70–80 ms after the stimulus onset using EEG [1], [6] and electrocorticography (ECoG) [24], and at later latency of 150 ms using MEG [25]. Even earlier attentional modulation between 20–50 ms has been observed both in EEG and MEG [2], [5]. Notably, the N100 response (N100m in MEG) at ∼100 ms from stimulus onset, generated at least partly in the primary auditory cortex [5], [8], was increased in amplitude during selective attention in all of these studies. Similarly with PET, selectively attending to left and right-ear tones was found to increase the overall activity in the contralateral auditory cortices and in frontal cortex [9], while the visual control task increased signal measured from the occipital visual areas. However, the dichotic listening task has some features making inference about the neurophysiological attentional mechanisms problematic, for instance, because of interaction between left and right side of the brain during binaural *vs.* monaural listening (see, *e.g.*, [26]).

When subjects are led to expect tones of a certain frequency, they tend to detect the expected frequency tones better than the ones with an unexpected frequency when using a continuous noise masker [27], [28]. The frequency range where perception is enhanced, ‘attentional band’, resembles the critical band and auditory filter measures obtained psychophysically [27]. However, when a bandpass-filtered noise burst is presented just before the target (‘gated noise masker’) the detection performance of unexpected tones is actually improved while the expected tone detection is worsened [29]. This would suggest that priming the cue with a gated noise masker expands the detection template, whereas a continuous masker masks the frequency areas adjacent to the target tone frequency without any special interaction with the target tone detection. When using a continuous notched-noise masker, the detection threshold is lower at the frequencies within the notch, near the notch center [30], and again higher at frequencies residing on the outer edges of the noise masker, close to notch corners [31].

In contrast to the evidence favoring a gain-based model of selective attention, increase in neuronal selectivity [17] and shifts in receptive fields towards attended location have been reported in the visual cortex [18]. Within the auditory modality, a recent combined fMRI and MEG study in humans suggested feature-specific enhancement of neural selectivity to phonetic *vs.* spatial features in the anterior secondary auditory cortex *“what” vs.* posterior secondary auditory cortex *“where”* processing pathways [21]. Furthermore, recent studies in ferrets have suggested task-dependent modulation of spectrotemporal receptive fields (STRFs) of primary auditory cortex neurons during classical conditioning [19]. Specifically, STRFs were characterized by playing the animals a number of spectrally complex auditory stimuli (‘temporally orthogonal ripple combinations’, TORCs) for 2.5 minutes and reverse-correlating the neural response with the TORC parameters. During an active tone detection task, the majority of STRFs were modulated in comparison to the passive STRF, enhancing the target frequency representation. The most common type of plastic receptive field modulation was excitatory field enhancement at the target frequency and lateral suppression that correlated with improved task performance [19], [32]. Further, in one third of the cells showing facilitation the receptive field modification was found to be quickly reversible (*i.e.*, the post-behavioral task passive STRF was highly similar to the pre-behavioral one, see also [32]), suggesting that these effects could be due to transient selective attention type of phenomena.

**Figure 1:**
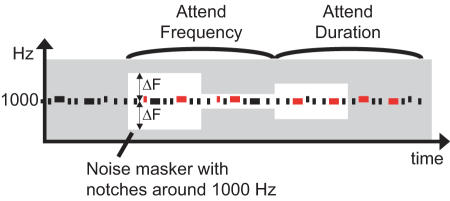
A schematic illustration of the task paradigm used. Target tones, indicated in red color, were either higher in frequency or longer in duration. The background grey represents the noise masker, while the white area represents the gaps in the noise.

**Figure 2:**
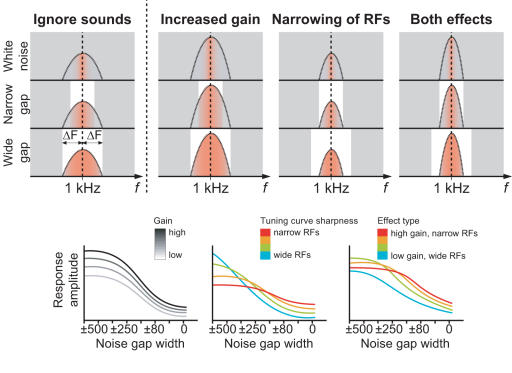
Illustration of the hypothesized effects of selective attention. Top: The bell-shaped curve represents the presumed single-neuron RFs during baseline (“Ignore”), and the proposed attention-dependent changes in the RFs (increased gain *vs.* narrowing of RFs *vs.* both effects). Here, the noise suppresses responsiveness of the neuron to the 1-kHz tone as a function of the overlap with the receptive field of the neuron. Thus, the red-coloured area below the bell-shaped curve indicates how likely the neuron will respond to the 1-kHz probe sound. In the white noise condition, only neurons optimally tuned to the tone respond [61]. Below: Hypothesized effects at the level of neuronal population responses as a function of notch width. The curves are only suggestive, based on a simple simulation (see Methods for details). Still, it should be noted with “gain only” mechanism, the amplitude reduction curve is assumed to stay identical between the stimuli endpoints, only scaled differently, while the other mechanisms would result in modulation of the basic shape of the amplitude reduction function as well.

Here, we quantified auditory cortex frequency tuning during selective auditory attention to frequency *vs.* duration *vs.* an ignore sounds task in 20 healthy subjects by masking 1000-Hz tones with continuous white noise band-stop filtered to contain frequency notches [33] of parametrically varying width around 1000 Hz (Figure 1). When the notch is narrower, the masker edges are closer to the 1-kHz standard tone in the frequency axis. Due to the tonotopic organization of the human auditory system, starting already from the cochlea, this induces stronger frequency masking and makes the detection of both standard and target tones more difficult by decreasing the effective signal-to-noise ratio. Therefore, we expected gradually smaller neural responses with narrower notches together with poorer task performance. We were specifically interested in estimating how attention influences the shape of the function describing how neural response diminishes with narrower notches. We hypothesized that an increase in neuronal gain would result in multiplicative increase of auditory-cortex response amplitude as a function of the width of the notch, still keeping the basic shape of amplitude reduction function the same, whereas enhanced selectivity would result in a more level amplitude reduction as a function of the notch width (Figure 2). This latter hypothesis is based on assumption of enhanced frequency selectivity in the auditory system narrowing, in our experiment, the receptive field near the 1 kHz. Thus, frequency masking would have less effect on the neural level, requiring narrower frequency notches to mask out the same amount of the 1-kHz tone than with broader receptive fields. A combination of these two effects was hypothesized to occur in the case that increased neural gain and enhanced feature selectivity together explain selective attention.

## Results

**Figure 3:**
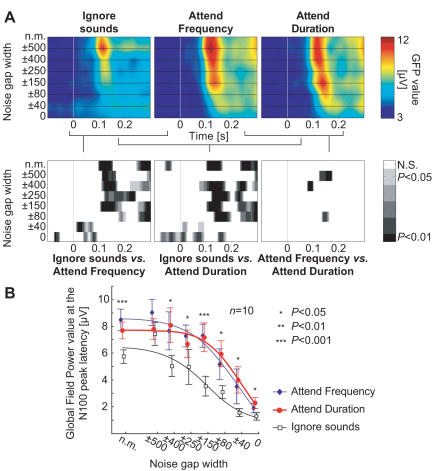
Global field power (GFP) changes as a function of the task. (A) Grand average GFP of electrical activity as a function of time *vs.* width of the noise gap (‘n.m.’ stands for ‘no masker’). Results of paired t-test comparisons between the conditions demonstrate the largest effects around the N100 peak latency and the similarity of the two Attend conditions. (B) Global Field Power amplitudes (±standard error of the mean) at N100 peak latency show the non-multiplicative suppression in amplitude with narrower notches during selective attention (Attended conditions *vs.* ignore sounds: * *P*<0.05, ** *P*<0.01, *** *P*<0.001)

**Figure 4:**
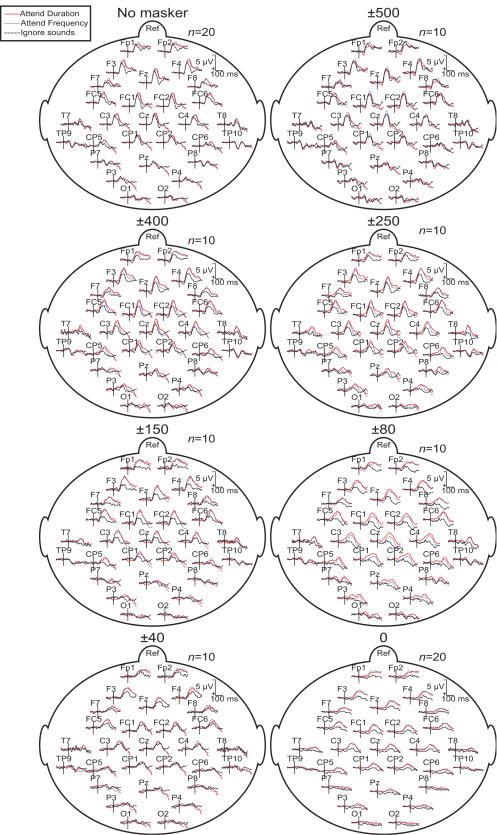
Grand average ERPs to the standard tone across all conditions and stimuli show the typical attention effect: N100 amplitude increase at ∼100 ms from stimulus onset during attention. Notably, the difference between attended and the ignore condition N100 amplitude is largest with narrow notch widths (e.g., ±150, ±80). The ERPs also demonstrate how the latency of N100 peak is delayed as frequency notch is narrower.

**Figure 5:**
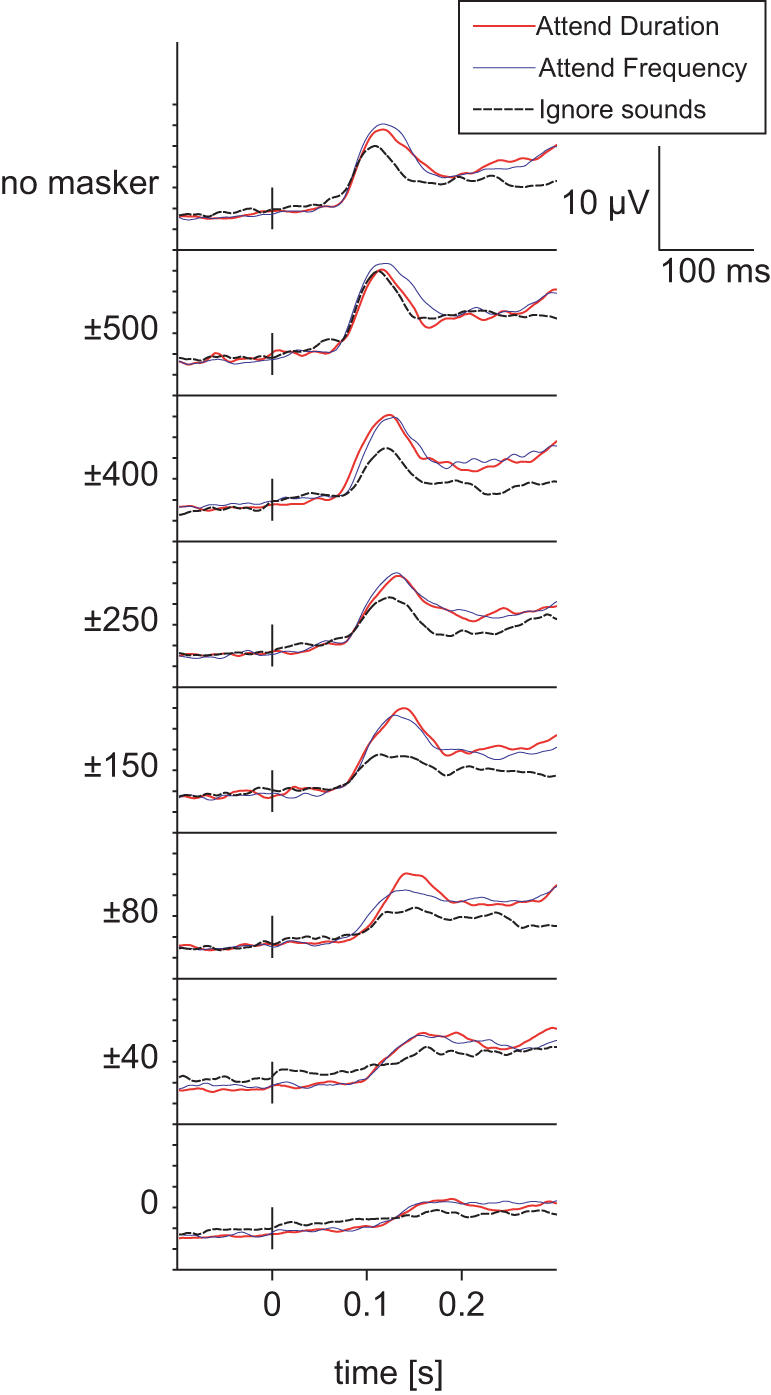
Grand average GFP measure of the evoked activity to the standard tone shows the same main effect as the ERPs in Figure 4 with a better signal-to-noise in narrow notch widths.

Due to frequency masking, the amplitude of the peak in global field power [34] (GFP) of event-related brain responses at 100 ms from stimulus onset (N100) decreased as an inverse function of the noise gap width (F_(7,63)_ = 10.69, *P* = 0.0020, ε = 0.24; see Figure 3B). The N100 event-related potential (ERP) was most prominent at the central and frontal electrode positions (Fz, Cz) due to our choice of reference electrode and binaural stimuli (ERP waveforms are shown in Figure 4 and the resulting GFP waveforms in Figure 5). Together with peak amplitude decrease the latencies increased as a function of the narrowing notch (F_(7,63)_ = 4.22, *P* = 0.013, ε = 0.45)

**Figure 6:**
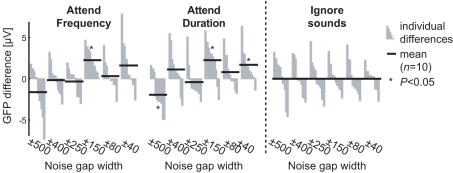
Comparison of predicted GFP amplitudes to the observed GFP amplitudes at different noise gap widths shows that the function of GFP amplitude reduction is significantly different during selective attention from that predicted by a pure gain mechanism. The grand average GFP amplitude function during Ignore task was fixed to the GFP amplitudes of end-point stimuli types during Attend tasks. This was done across all subjects in order to obtain prediction of GFP amplitude for the in-between stimulus types. Positive value means that the observed value was bigger than the prediction. The vertical grey bars denote subject-wise differences, sorted by size, while the horizontal black bar is the average GFP difference (n = 10 for each stimulus type). The values during Ignore task are used only as a reference, as they are zero-mean due to the comparison algorithm.

Selective attention significantly increased the GFP amplitude at the N100 peak latency (main effect: F_(2,18)_ = 49.20; *P*<0.00001, ε = 0.85; Attend *vs.* Ignore contrast: F_(1,9)_ = 93.76, *P*<0.00001), but as a non-multiplicative function of the width of the noise masker notch (see Figure 3B). Specifically, after fixing the grand average GFP amplitude reduction function during Ignore task to the end-point stimuli types (no masker, white noise) of Attend conditions with a simple linear scaling and offset, a comparison was made between the predicted GFP amplitudes for the notched-noise stimuli types (±500, …, ±40 Hz) and the observed GFP amplitudes, revealing a significant *Datatype* (*i.e.*, predicted *vs.* observed)×*Stimulus* interaction effect (F_(2,18)_ = 5.14; *P* = 0.014, ε = 0.56) and nearly significant *Datatype*×*Condition*×*Stimulus* interaction (F_(10,90)_ = 39.00; *P* = 0.129, ε = 0.42), which both reached significance by taking the Attend *vs.* Ignore contrast (F_(1,9)_ = 17.18; *P* = 0.0025 for the first interaction, F_(1,9)_ = 5.57; *P* = 0.043 for the second). To further assess this effect, the observed GFP peak amplitudes were pairwise compared to the predicted ones. With noise gap ±500 Hz, the observed value was smaller than predicted, and with noise gaps ±150 and ±40 Hz larger than predicted (Figure 6). These findings suggest that increased gain and enhanced selectivity together explain selective attention (as hypothesized in Figure 2). The peak latencies were also significantly longer during selective attention (main effect: F_(2,18)_ = 115.35, *P*<0.00001, ε = 0.76; Attend *vs.* Ignore contrast: F_(1,9)_ = 134.47; *P*<0.00001), tentatively suggesting that the enhanced feature selectivity could be accomplished through inhibitory mechanisms [35].

**Figure 7:**
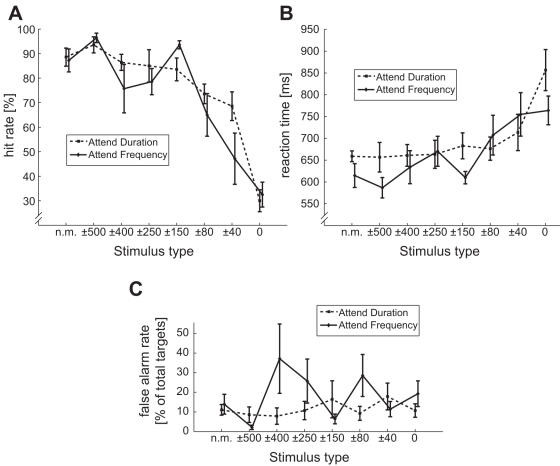
Due to frequency masking, the auditory responses to both standard and target tones decrease with narrower notches (responses to standard tone shown in Figures 4 and 5). Concordantly, the detection of target tones becomes more difficult, as shown by (A) hit rate decrease and conversely by (B) reaction time increase (‘n.m.’ stands for ‘no masker’). (C) The false alarm rate did not change as a function of the stimulus.

There were significantly fewer correct responses (F_(7,63)_ = 30.30, *P*<0.00001, ε = 0.33, Figure 7A) and longer reaction times (F_(7,63)_ = 8.82; *P*<0.001, ε = 0.42, Figure 7B) with narrower notches. Hit rates in the Attend Frequency *vs.* Attend Duration conditions did not differ significantly, indicating that the two discrimination tasks were of similar difficulty. Hit rates and reaction times correlated significantly with the GFP amplitude at the N100 peak latency (Table 1).

**Table 1:** Behavioral data from Attend conditions (data across all stimuli were pooled together).

Condition	Behavioral measure	x̄±SEM	*r*	*p* (uncorrected)
Attend Duration	HR [%]	76.2±2.7	0.54	0.00000030
	RT [ms]	696±13.1	−0.30	0.0073
	FA [% of targets]	11.6±1.8	0.30	0.029
Attend Frequency	HR [%]	72.0±3.3	0.56	0.00000015
	RT [ms]	666±13.7	−0.55	0.00000026
	FA [% of targets]	18.1±3.3	−0.03	0.83

Correlation values of GFP at N100 peak latency *vs.* hit rates (HR), response times (RT) and false alarms (FA) show how the detection of targets and neural response strength was linked.

## Discussion

Our experimental data corroborate the previous findings of attentional augmentation of the N100 response. Our novel procedure was to focus on the general shape in which the N100 amplitude changes as a function of parametrically varying, continuous notched-noise masker. The shape of this function (Figure 2 *vs.* 3B, see also Figure 6) suggests that multiplicative gain increase alone cannot explain the present findings, but rather that a combination of increased gain and enhanced selectivity underlies auditory selective attention, similarly to what has been recently suggested to be the case in the human visual system [20], [36]. This is analogous to the monkey visual system, where selective attention increases response gain of orientation-selective neurons in the early areas MT, V1 and V4 [37]–[40]. Some of these animal studies report only the gain increase, that is, no difference was found in the preferred orientation that the neurons responded to [37], [38]. Still, more recent studies propose a combination of increased gain and selectivity to explain the observed modulation in areas V4 and MT [39], [40] or an additional ‘response bias’ towards higher-contrast stimuli in case of multiple stimuli inside the neurons’ receptive field [41].

In the present study, attentional modulation was strongest with the intermediate notch widths (*e.g.*, ±150 Hz and ±80 Hz; see Figures 3 and 4), again decreasing in amplitude with ±40 Hz notch and plain white noise. As the task difficulty increased with decreasing notch, resulting in poorer performance, we could tentatively say that task difficulty (or attentional load) did increase the proportional attentional effect or sensory gain, but only to a certain extent, which could even be related to the physiological limits of the human auditory system (the critical band). The increase in attentional effect together with task load is in line with previous studies [24], however, it should be noted that in our study the presented tones were deliberately adjusted to be perceptually very weak, and the use of random, long inter-stimuli interval (ISI) made the task even more difficult.

Here, we did not specifically address the question of where these attentional modulations took place. Some previous studies have suggested that attention-related modulations in humans are confined to secondary auditory cortex [15], while others have suggested involvement of the primary auditory cortex [5], [8], [24]. A recent study in awake monkeys employing a masking paradigm similar to the present study found neuronal population responses in A1 [42] that were clearly more sharply tuned than in noninvasive measurements of the auditory cortex activity [43], [44] (including the present study). This suggests that noninvasive human measures of tuning also encompass activity of less-sharply tuned neurons in non-primary cortical areas. An interesting lesion study shows that auditory attention strongly modulates non-primary auditory areas and can lead to perception of sound onset and offset, termed ‘deaf-hearing’, even with an absence of primary auditory areas [45]. This could be explained by by top-down attentional control signals, projecting from the prefrontal cortex to the remaing non-primary auditory areas, leading to conscious perception. Therefore, caution should be exercised when drawing conclusions in which specific auditory cortical areas the present attention effects are generated.

We quantified the EEG response by a well-established technique of using the tone-evoked N100 peak amplitude. This, of course, does not completely define the neural activity during perception, because the evoked potential is a product of massively synchronized neural populations, and makes hypotheses about underlying neural mechanisms only speculative. Due to this, the increase in the observed EEG amplitude during Attention could be accounted for by an increase in synchrony of the neural activity (*i.e.*, less jitter in single epochs). Still, this type of enhancement alone would produce results roughly identical to the ones with multiplicative gain increase (see Figure 2). The question of linearity between the EEG response and stimulus level might also need clarification. The N100m response (MEG counterpart of N100) amplitude to a simple tone increases approximately linearly with level only very near the psychophysical threshold, showing saturation at higher levels [46]. The stimulus level dependence of the evoked potential amplitude can be even more complicated with spectrally complex stimuli as the ones used here, where we employed frequency masking to modify the perceived stimuli level. However, as we used identical auditory stimuli across all conditions, we did not need to assume linearity between the response amplitude and perceived stimulus level when analyzing the main results (although the assumption of linearity was made in the simulations, so direct comparisons between the curves shown in Figure 2 and Figure 3B should be done cautiously). Selective attention can naturally also alter this linear or nonlinear relationship between the EEG amplitude and the stimulus level, but we did not specifically address this question here.

**Figure 8:**
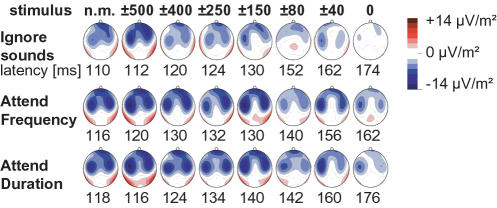
EEG current source density (CSD) maps (‘n.m.’ stands for ‘no masker’ stimulus). The topography of CSD maps at the N100 peak latency was highly similar across the ignore and selective attention conditions.

According to some theories, selective attention modulates neural populations separate from those processing the stimulus *per se* [22] so that there are in fact two distinct auditory pathways: one faithfully transmitting the incoming sensory information and another attention-modulated pathway analyzing the task-relevant acoustic features [15]. However, given that the current source density maps disclosing left and right hemisphere auditory cortex sources were observed to be highly similar in the present study across conditions (see Figure 8), our findings are in favor of models and previous results suggesting a direct modulation of feature-specific neurons and their receptive fields [5], [6], [19], [24], [32].

Interestingly, attending to sound duration produced effects at the N100 latency similar to attending to sound frequency. Psychophysical studies indeed demonstrate that the template used in detecting an auditory stimulus stores both its frequency and duration [47]–[49] (*i.e.*, duration is not represented independently of frequency).

While our results indicate that increased sensory gain alone cannot explain selective attention in the auditory cortex, but that enhanced frequency selectivity in the underlying tonotopically organized neurons additionally contributes to filtering of task-relevant stimuli from noise, there are number of alternative mechanisms that could explain enhanced selectivity during selective attention. One possible mechanism could be the receptive field modulations, evidenced by neurons' best frequency (BF) shifts towards the target sounds in lower animals [19], [50]. These effects have been observed to take place on the timescale of seconds or minutes when induced by electric stimulation [19], [51] (however, see also [21]). It is also possible that such receptive field modulations could be explained by modulation/tuning of sound processing at subcortical [52], or even cochlear [53], [54], level, driven by corticofugal connections from the primary auditory cortex [51], [55].

Alternatively, the increased selectivity can be explained by lateral interaction mechanisms, which have been shown to occur in early vision due to attention [56]–[58] and in human audition following exposure to notched-noise stimuli [31], [59]. The longer response peak latencies in the present study during the selective attention conditions provide tentative support for inhibitory mechanisms, but as previous studies have found opposite latency effects during attention (*e.g.*, [14]), this finding by itself should be interpreted carefully. Importantly, the changes in the balance of excitation and inhibition could explain the observed relatively higher amplitude with ±500 Hz notch during the Ignore task (see Figure 3B). This observation should not be considered as a measurement artifact, since in a previous study with similar symmetrical notches around the 1-kHz tone and passive reading task, the MEG dipole amplitude was found to be largest with about ±400–500 Hz notches, and decreased in amplitude with wider notches (±600–1000 Hz) [43]. It is, for instance, possible that when increasing the notch width around the test tone frequency, the lateral inhibitory band of the sharp notched-noise edge at a certain notch width reaches neurons which normally inhibit neurons whose CF is near the target tone frequency, consequently allowing more neurons to respond to the test tone. Further neurophysiological studies are, however, needed to address the question of exactly which neural mechanism underlies the enhancements in frequency selectivity.

### Conclusion

The current experiment shows that auditory selective attention in humans cannot be explained by a gain model, where only the neural activity level is increased, but rather that selective attention additionally enhances auditory cortex frequency selectivity. Selective attention increased the amplitude of the evoked brain responses with all of the used notch widths, but as a non-multiplicative function of the width of the notch when compared to the Ignore condition. The question of exactly which neural mechanism increases the selectivity still requires further studies, but we hypothesize that a combination of both increase in cortical gain and enhanced selectivity could best explain the results. Additionally, based on the current data, lateral inhibition likely plays a role in enhancing the auditory cortex response.

## Materials and Methods

### Subjects and stimuli

20 healthy right-handed volunteers (13 males and 7 females, age 18–28 years) with normal hearing participated in the study. The studies were carried out in accordance with the Helsinki Declaration, ethical approval was obtained from the Coordinating Ethics Committee of the Hospital District of Helsinki and Uusimaa and a written voluntary consent was obtained from each subject prior to participation. The stimulus paradigm used was highly similar to one that we have previously used to quantify auditory-cortex sound frequency tuning [43]. Within a given block, repetitive 100 ms 1000 Hz sine wave tones (65 dB SPL, inter-stimulus interval (ISI) 1.5–2.5 s) were presented either in silence, during continuous white noise, or during white noise band-stop filtered (120 dB/Hz) with one of six different notch widths ±ΔF (±500, ±400, ±250, ±150, ±80, and ±40 Hz) centered at 1000 Hz. The SPL of the noise maskers was adjusted before the experiment in 1-dB steps [60] so that the 1000-Hz tones were at 50% hearing threshold with the white noise masker (range: 47–53 dB SPL). To keep the length of the sessions within reasonable limits, half of the notched-noise maskers (±500, ±150, and ±40 Hz) were presented to 10 of the subjects, and the rest of the noise maskers (±400, ±250 and ±80) to the remaining 10 subjects. Occasionally, the 1000 Hz tones were replaced by infrequent target tones either higher in frequency (5%, 1020 Hz), longer in duration (5%, 150 ms), or both higher in frequency and of longer duration (5%). All tones had 5-ms linear rise and fall times. The auditory stimuli were presented using a PC and Presentation Software (Neurobehavioral Systems Inc., Albany, CA, USA). All of the sound files used in the experiment were created using Matlab (R12.1, MathWorks Inc., Natick, MA, USA) with 44.1-kHz sampling frequency and 16-bit precision. The sounds were presented binaurally through two high-quality speakers (Roland Stereo Micro Monitor MA-8), which were located symmetrically on both sides of the computer monitor (distance from subjects' head one meter).

In addition to the baseline condition, where the subjects were instructed to watch a silent movie and ignore the auditory stimuli, there were two discrimination conditions, where the subjects had to press a button with their right index finger in response to detection of frequency deviants (Attend Frequency), and duration deviants (Attend Duration). During these tasks, a small fixation cross was displayed on the computer screen. Identical auditory stimuli were presented during all three tasks. A hit was defined as a button press before the onset of the next stimulus. Late responses and responses to non-target stimuli were regarded as false alarms.

### Data acquisition and analysis

The 32-channel EEG was recorded (0.1–225 Hz passband, 500 Hz sampling rate; Brain Products GmbH, Germany) in a sound-attenuated and electrically shielded room. 800-ms epochs with 200-ms pre-stimulus baseline were filtered with a 0.5–40 Hz passband and averaged relative to the onset of the 1000-Hz sounds, with responses exceeding ±75 µV at any channel rejected due to possible extra-cerebral artifacts. The number of accepted epochs per stimulus type and condition (*e.g.*, a given noise-masker type during Attend Frequency condition) was on average 108 (out of 119).

After averaging and obtaining the event-related potential (ERP), the Global Field Power (GFP) measure [34] was calculated for each epoch with a formula
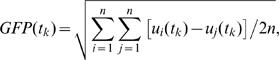
(1)where *u_i_*,*u_j_* are the evoked potentials from channel numbers *i*,*j* at each time point *t_k_*, and *n* is the number of electrodes used. The GFP peak latencies for N100 response at around 100 ms were determined for each stimulus type and condition from the grand averaged GFP. Mean GFP amplitude of ±20-ms time window around this grand average peak latency was then used as the N100 peak value for each subject. The mean GFP value during pre-stimulus period (100–0 ms) did not differ significantly across stimuli or conditions, so it was subtracted from the N100 peak; the resulting value was used as the individual GFP value in further analysis. Scalp current densities were also calculated for each stimulus type and condition, to reveal possible changes in source configuration across conditions. Behavioral and neurophysiological data were analyzed using a two-way, repeated measures analysis of variance (ANOVA), with Condition (Attend frequency, Attend duration, Ignore sounds) and Stimulus (no masker, ±500, …, 0) as within-subjects factors. To test the hypothesis that function of amplitude reduction was modified during attention, an additional Datatype (observed data *vs.* predicted data) within-subject factor was used. The reported *p*-values from the ANOVA were corrected using Greenhouse-Geisser correction for degrees of freedom, but are reported with original, uncorrected degrees of freedom.

### Simulation of hypothesized effects

The simulation of different effects of attention to the neural response amplitude (depicted in Figure 2) was done in Matlab (R12.1, MathWorks Inc., Natick, MA, USA). There, an array of neuron-like elements was created, each with own characteristic frequency (CF) and tuning curve around it. The basic shape of the tuning curve was a single-parameter rounded-exponential *Roex(p)* filter
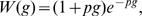
(2)where *p* is the steepness of the filter and *g* the relative width of the frequency notch. Here, the *Roex(p)* filter shape was chosen only for simplicity and *g* was used as a factor of difference between each neurons CF and the 1-kHz probe sound. Neurons' firing probability at each time point during stimulus presentation was inversely proportional to the tuning curve shape. The time course of this hypothetical ‘neural population’ was calculated in 1-ms steps, during which each neuron was either ‘on’ or ‘off’, and the population response amplitude was defined as number of active neurons during stimulus presentation *vs.* baseline.

The effect of multiplicative gain was simulated by increasing the firing probability for each neuron (*i.e.*, lower threshold of activation for all the simulated neurons). The tuning curve sharpness change was simulated by changing the *p* parameter in *Roex(p)* auditory filter, uniformly across all simulated CFs (higher *p*, sharper tuning curve). In addition to the isolated changes, a combination of effects was simulated by both increasing the gain and sharpness.
